# Semen quality, hormone profile and histological changes in male albino rats treated with *Corchorus olitorius *leaf extract

**DOI:** 10.22038/AJP.2019.13426

**Published:** 2019

**Authors:** Daniel Orieke, Obioma Christopher Ohaeri, Ifeoma Irene Ijeh, Solomon Nnah Ijioma

**Affiliations:** 1 *Department of Biochemistry, College of Natural and Applied Sciences, Michael Okpara University of Agriculture, Umudike, Abia State, Nigeria*; 2 *Department of Zoology and Environmental Biology, College of Natural and Applied Sciences, Michael Okpara University of Agriculture, Umudike, Abia State, Nigeria*

**Keywords:** Corchorus olitorius, Anti-fertility, Hormonal, Sperm sample, Histology

## Abstract

**Objective:**

In this study, the anti-fertility effect of *Corchorus olitorius* leaf extract (COLE) was evaluated in adult male rats.

**Materials and Methods:**

Forty rats assigned to 4 groups of 10 rats each, were orally given COLE for 28 days. Group 1 was the control group but groups 2, 3 and 4 were administered with 250, 500 and 1000 mg/kg body weight, respectively and were considered the test groups. Blood collection from the animals was performed at the end of treatments and blood samples were used for reproductive hormone assays. Also, sperm sample quality was ascertained, and basic organs were evaluated histologically for all groups.

**Results:**

A significant fall in relative organ weights for the testes and prostate was observed following high-dose treatment (p<0.05). Sperm sample pH, and individual sperm motility, viability, progressiveness and concentration decreased, while total abnormalities increased following high-dose (1000 mg/kg) treatment (p<0.05). Serum concentration of FSH significantly increased at 500 and 1000 mg/kg dose levels while LH and testosterone concentrations were significantly higher than control at all dose levels (p<0.05) except estrogen which was higher than control at 250 and 500 mg/kg but lower at 1000 mg/kg dose level. Control testes showed intact histological architecture with mature spermatid density of 300 cells per tubule and well differentiated Leydig cells, while those animals treated with 250 and 500 mg/kg of the extract, were without significant pathology but had average spermatid densities of 200 and 280 cells per tubule, respectively. Significant azoospermia and spermatid density of only 30 cells per tubule and prostatic degeneration were seen in the group treated with 1000 mg/kg body weight of the extract.

**Conclusion:**

Consumption of high amounts of *C. olitorius* may inhibit reproductive functions in males.

## Introduction

With a global incidence rate of about 30%, infertility among males is fast becoming a matter of global concern with males reported to be directly involved in about 50% of global infertility prevalence. Understanding the prevalence and nature of male infertility across Africa has been quite difficult because of insufficient data and the fact that African males rarely agree to undergo fertility tests and usually prefer to blame the females for nearly all cases of infertility in the family (Agarwal et al., 2015). Male infertility among Nigerians has also been reported to be responsible for about 20-50% of all infertility cases in different parts of the country (Eze, 2012). A number of factors that contribute to male infertility includes poor semen quality (Willott, 1982), hypothalamic pituitary disease, testicular disease, disorders of sperm transport and idiopathic male disease (Kaundal et al., 2016). Other known causes are exposure of the testicles to abnormally high temperatures, developmental history such as cryptorchidism, erectile dysfunctions, diseases such as diabetes and respiratory infections, and past surgical and cancer treatments. Other contributors are lifestyle factors such as exposure to environmental toxins like cadmium, mercury, arsenic compounds, hydrocarbons, alcohol, cigarette smoking and pesticides (Hideyuki et al., 2012). 

Global rise in infertility cases amongst male, has been attributed to decline in semen quality (Abarikwu, 2013). About 2% of all men have sperm parameters below optimal values. Infertility may be caused by low sperm count, inadequate sperm motility, abnormal morphological structures or a combination of these factors (Kumar et al., 2015). Decline in or inappropriate secretion of male sex hormones is also another major contributing factor. Although male infertility due to endocrine disorders reportedly accounts for below 3% of infertility cases in males (Hideyuki et al., 2012), hormone assay for males is done to both identify causative factors for these endocrine abnormalities and to obtain prognostic information. Agents that interfere with reproduction may do so by affecting spermatogenesis at the testicular level or changing the body’s hormone profile or both.

Numerous plants are historically known for their ability to negatively affect male fertility. Jahan et al. (2009) also reported the anti-fertility effects of some tested medicinal plants. Anti-fertility effects of plants may be due to ecobolic, estrogenic and spermicidal properties or rise from the toxic effects of the extract on the reproductive organs (Marles and Farnsworth, 1994). Some plants were reported by Choudhary et al. (1990) to have significant anti-fertility effects include *Diospyros embryopteris*, *Melia azedarach* (100% abolition of libido in males) and *Casiarea tomentosa* (60% abolition of libido in males).

*Corchorus olitorius* is one of the plants that were recently evaluated for anti-fertility properties*. *The plant belongs to the genus *Corchorus* which belongs to the family Tiliaceae, comprising of about 60 different species disseminated worldwide with 30 enumerated in Africa. *C. olitorius *is a leafy vegetable commonly called jute mallow in English, “*Ewedu*” and “*Ahihara*” in the southwestern and southeastern parts of Nigeria, respectively. *C. olitorius* is generally known as “Jute” and is a vegetable popularly grown in places like the tropical African countries, Malaysia, South America and the Caribbean (Onyeka et al, 2007; Ujah et al, 2014).* C. olitorius* leaves are used both as food and a herbal medicine in several countries of the world including India and Philippines. Some of the ethno-medicinal uses of the plant’s leaves include, treatment of pain, fever, chronic cystitis and tumor growths. The seeds are said to possess estrogenic activity and polyphenols isolated from the plant are known to have anti-obesity effects (Oyedeji and Bolarinwa, 2013).

According to a study done by Oyedeji et al. (2013), treatment of rats with aqueous leaves extract of *C. olitorius* lowered testosterone levels, and motility, concentration and viability of sperm but increased the proportion of sperm cell abnormalities (with distortion of testicular structure) relative to their individual control groups. We also reported in our previous work that long-term administration of *C. olitorius* leaves extract at high doses may be deleterious to health due to its toxic effects on the blood and some organs (Orieke et al., 2018).

In this work, we evaluated the anti-fertility effects of methanolic extract of *C. olitorius *leaves in male albino rats to provide more knowledge on the extent to which *C. olitorius *leaves may affect fertility in males considering the fact that literature in this regard is quite scanty. This study presents protocols and results of a wider and deeper evaluation of the anti-fertility effect of the methanol extract covering areas like hormone assay (for FSH, LH, estradiol and testosterone levels), sperm analysis, testicular and prostatic histology for all doses of treatment and effect on body/relative organ weights against other literature sources which focused on few parameters and on the water extract of *C. olitorious* leaves. Moreover, it is established that extraction solvents affect the quality, quantity and pharmacology of plant extracts and extraction with methanol is known to give better extract yield than water (Do et al., 2014). 

**Figure 1:**
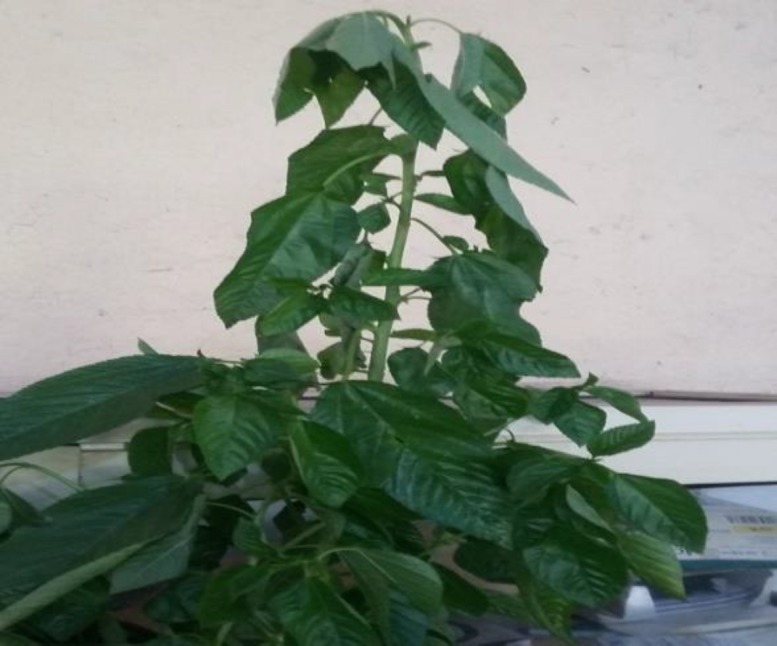
Fresh leaves of *Corchorus olitorious* harvested from Umudike environment, Ikwuano Local Government Area, Abia State, Nigeria

## Materials and Methods

**Collection and authentication of the plant material**

Fresh leaves of *C. olitorius *were collected from Umudike environs in Ikwuano Local Government Area of Abia State, Nigeria. The plant materials were identified by a Taxonomist and Forest Manager in the Department of Forestry, College of Natural Resources and Environmental Management, Michael Okpara University of Agriculture, Umudike. A voucher number MOUAU/VPP/17/009 was assigned to a sample which was preserved in the herbarium of the Department of Physiology and Pharmacology in the same University. An image of *C. olitorious* sample collected from Umudike environment, Ikwuano Local Government Area, Abia State, Nigeria is presented in Figure 1.

**Preparation of plant extract**

The collected fresh leaves were dried in the shade at 25^o^C for two weeks and thereafter, pulverized in a locally fabricated milling machine. One hundred (100) grams of the pulverized material was packed into the material chamber of the Soxhlet extractor and extracted by methanol at a specific temperature (60^o^C) for 48 hr. At the completion of extraction, the solvent in the extract was evaporated at 40^o^C in a hot air oven to obtain a crude extract which weighed 8.18 g, representing a yield of 8.18%. The extract was preserved in the refrigerator until needed and is hereafter referred to as *C. olitorius *leaf extract (COLE). 

**Animals used for the experiment**

Forty sexually mature male Wistar rats (10-12 weeks old) obtained from the laboratory animal house of the College of Veterinary Medicine, Michael Okpara University of Agriculture, Umudike, were used. The rats were assigned to 4 groups of 10 rats each, in metal cages and they had free access to food and water at will. An acclimatization period of 14 days was considered for all the rats before the commencement of experiment. The experimental protocol of the study was approved by the animal ethical committee of the Department of Physiology and Pharmacology, Michael Okpara University of Agriculture, Umudike, in accordance with the guidelines for care and use of laboratory animals established by the National Institute of Health. Design of the experiment was such that group 1 rats were administered normal saline only and served as the control group while groups 2, 3 and 4 were administered with COLE 250, 500 and 1000 mg/kg body weight, respectively orally for a sub-acute period of 28 days. The choice of these doses was based on rational decision and to maintain a non-lethal dose based on findings from acute toxicity evaluation of the extract as reported in our previous study (Orieke et al., 2018). The rats were all sacrificed on the 29^th^ day for blood collection and sera obtained from the blood were analyzed for their reproductive hormone content. Sperm sample collected from the caudal epididymis of each rat was also analyzed for qualitative and quantitative characteristics. The animals’ initial and final body weights and food intake were all measured and recorded. The testes, epididymis and prostate were harvested from each animal, weighed and preserved in 10% formalin for histological study. Relative organ weight (ROW) for each organ collected, was ascertained using the expression: 

$\mathrm{Row}=\frac{\mathrm{Organ weight}}{\mathrm{Body weight}}\times \frac{100}{1}$

**Quantitative intra-assay hormone analysis**

The concentrations of follicle stimulating hormone (FSH), luteinizing hormone (LH), testosterone and estradiol in each serum sample were determined by the chemiluminescence intra immunoassay techniques using kits and in accordance with standard protocols outlined by the kits producer AUTOBIO DIAGNOSTICS CO., LTD. Zhengzhou, China (Zade et al.,2013).

**Sperm samples collection and analysis**

Each rat was euthanized by cervical dislocation and its epididymis was harvested. Sperm samples were collected from the epididymal reserve at the caudal portion of the epididymis and a smear of same was prepared on the preheated glass slides for evaluation. 

**Sperm samples color and consistency**

The color and consistency of the sperm samples were evaluated macroscopically by mere observation and recorded. The consistency scale (1-4) adopted by Chibundu (2013) was used. Adopted color scale (1-3) was: 1 (white), 2 (milky white) and 3 (creamy white). For consistency, the scale was 1 (watery), 2 (slightly thick), 3 (thick) and 4 (very thick).

**Sperm motility**

The method described by El-Sherbiny (1987) was adopted. The sperm samples collected via epididymal washings from each treatment group, was evaluated for progressive motile sperm cells immediately after collection. A smear of one drop of the sperm sample was made on a preheated glass slide and viewed under light microscopy at a low magnification of X10 and X40 and scored subjectively in percentage. Only sperm cells moving in straight forward direction, were included in the motility count. Those moving in circles, backward direction or showing pendular movement were excluded. Individual sperm motility was scored based on the following motility scale: 1 (vigorously progressive), 2 (progressive), 3 (cycling movement) and 4 (stationary movement).

**Sperm viability (live proportion)**

The proportion of sperm cells that were viable (alive) was determined by staining a drop of the collected sperm sample with Eosin-Nigrosin stain. The stained-glass slide was allowed to dry for 30 sec before being fixed by ethanol and viewed under a light microscope at X100 magnification (oil immersion), and the proportion of the sperm cells that were viable, was counted using a hand-held stopwatch manual counter. A total of 300 sperm cells were counted and the number of viable ones was expressed as a percentage of the total number counted. The sperm cells that were alive (viable) did not pick the stain while those that were dead, did (El-Sherbiny, 1987).

**Sperm concentration**

This was determined in a haemocytometer in accordance with the method described by Ukar et al. (2016). A dilution of 1: 200 was made using a red blood cell pipette. Here, 10% buffered formalin solution was used as the semen diluting fluid to immobilize the sperm cells. The haemocytometer was then charged with a drop of the sperm solution and allowed for 2 min on a wet paper (for sperm cells to settle) before mounting on a light microscope stage and viewed at X40 magnification.

Sperm concentration per ml = No. of cells counted ×Dilution Factor×0.04 × 10^6 ^(Egbuka, 1995).

**Abnormal sperm proportion**

The percentage of abnormal sperm proportion was determined by the method described by El-Sherbiny (1987). A drop of the sperm sample was stained with Eosin-Nigrosin stain and the mixture was smeared on a glass slide and viewed under a lower magnification of X40 to check for primary and secondary abnormal sperm cells. Percentages of the differential abnormalities such as head abnormalities, tail abnormalities, mid-piece abnormalities etc. were also determined (El-Sherbiny, 1987).

**Histological examinations**

This was carried out in accordance with the method used by Ajonuma et al. (2005). Organs (testis, epididymis and prostate) preserved in 10% formalin, were processed into slides for histological examinations. Before embedding in paraffin wax, the tissue samples were dehydrated in graded ethanol. The embedded tissues were then processed using KD-TS6A tissue processor. Sections (5 µm thick) were cut using a Shandon Finesse Manual Rotary Microtome, model 325, Thermo-scientific, and dried onto super frost microscope slides (Fisher Scientific, Pittsburgh, PA, USA). For hematoxylin and eosin (H&E) staining, slides were dewaxed in xylene and dehydrated in graded alcohol and stained for light microscopy. Images were captured using a digital microscope attached to a computer.

**Statistical analysis**

Results are expressed as means±standard error of mean (SEM). Statistical analysis was done using one-way analysis of variance (ANOVA). Significant differences were assessed at 95% level of significance between control and COLE-treated groups using Duncan and LSD (*Post hoc*) tests. p values less than 0.05 were considered significant. Computer software package, SPSS version 21 was employed.

## Results

**Food intake, body weights and relative organ weights of rats treated with COLE**

Mean daily food intake significantly decreased with increasing dose of COLE when compared with control (p<0.05). Body weight gain in all test groups also decreased in a dose-dependent manner when compared with control (p<0.05) (Table 1). Relative organ weights (epididymis) was not significantly altered (p>0.05). Relative organ weights for the testes and prostate were significantly lowered only in the group treated with COLE 1000 mg/kg (p<0.05) while test groups administered with lower doses had values which did not significantly differ from those of the control group (Table 2).

**Table 1:** Effect of COLE on food intake per group and body weight changes

**Treatments**	**Mean daily food intake per group (g)**	**Day 1 body weight ** **(g)**	**Day 28 body weight ** **(g)**	**Body weight gain (g)**
Normal control	197.32±5.97	143.93±11.90^*^	153.97±15.70^*^	10.04±4.81
COLE 250 mg/kg	189.05±4.86	138.50±3.44	146.41±5.80^*^	7.91±3.38
COLE 500 mg/kg	186.22±6.90	159.48±7.87^*^	165.65±11.56^*^	6.17±5.24
COLE 1000 mg/kg	167.58±6.04	158.56±8.41^*^	160.70±10.03	2.14±2.62

Results are expressed as Mean±SD. Means marked with * in the same column are significantly different from the normal control for p<0.05, n=10. COLE: *Corchorus olitorius* leaf extract.

**Table 2:** Effects of COLE on relative organ weights

**Parameters**	**Normal**	**COLE (250 mg/kg)**	**COLE (500 mg/kg)**	**COLE (1000 mg/kg)**
Epididymis (%)	0.24±0.03	0.22±0.02	0.21±0.02	0.21±0.02
Testis Index (%)	1.38±0.04	1.31±0.16	1.22±0.20	0.63±0.02^*^
Prostate (%)	0.002±0.00	0.002±0.00	0.002±0.00	0.00±0.00^*^

Results are expressed as Mean±SD. Means marked with * in the same row are significantly different from normal control for p<0.05, n=10. COLE: *Corchorus olitorius* leaf extract.

**Effects of COLE** **on sperm sample quality**

The color of sperm samples in all test groups was creamy white and did not differ from that of the controls. The pH values of test animals in the 500 were significantly higher than control while that of the 1000 mg/kg group was lower (p<0.05). Individual sperm motility scores were also higher in the COLE-treated groups than control, though a slight decline was observed in the highest dose group. Progressive movement of sperms also significantly decreased in the 1000 mg/kg body weight treatment group (p<0.05) but was not significantly altered in the 500 mg/kg treated rats (p>0.05). Sperm viability in the COLE treated groups were significantly lower than that of the control group (p<0.05) with higher severity in the highest dose group, where sperm concentration was also significantly lowered (Table 3). Total sperm abnormalities were also found to be higher in the COLE-treated groups with the 1000 mg/kg treatment group having the highest percentage abnormality of 14.83±0.81% when compared with the control value of 0.56±0.11% (Table 4). 

**Table 3:** Sperm viability, motility and concentration

**Parameters**	** Normal **	**COLE 250** **mg/kg**	**COLE 500** **mg/kg**	**COLE 1000** **mg/kg**
Sperm sample colour	2.00±0.00	2.00±0.00	2.00±0.00	1.80±1.03
Sperm sample pH	6.98±0.20	6.94±0.23	7.14±0.10^*^	6.65±0.08^*^
Individual sperm motility	1.00±0.00	1.40±0.52^*^	1.40±0.52^*^	1.30±0.82^*^
Progressive motile sperm	78.06±12.74	50.54±24.52^*^	68.69±5.24	42.68±17.50^*^
Viable sperm	88.33±1.03	73.78±6.02^*^	75.88±1.25^*^	66.39±3.61^*^
Sperm conc.	83.93±12.19	79.95±16.31	81.18±7.09	67.41±14.28^*^

Results are expressed as Mean±SD. Means marked with * in the same row are significantly different from normal control for p<0.05, n=10. COLE: *Corchorus olitorius* leaf extract.

**Table 4:** Sperm morphology and abnormalities

**Parameters**	**Normal**	**COLE (250 mg/kg)**	**COLE (500 mg/kg)**	**COLE (1000 mg/kg)**
Headless sperm (%)	0.10±0.11	0.27±0.18^*^	0.18±0.05	3.01±0.13^*^
Broken sperm (%)	0.14±0.01	0.34±0.28^*^	0.11±0.04	0.23±0.15
Bent midpiece (%)	0.00±0.00	0.03±0.04^*^	0.00±0.00	0.01±0.01
Twisted tail (%)	0.03±0.06	0.74±0.85^*^	0.07±0.08	2.38±0.32^*^
Tailless sperm (%)	0.02±0.03	0.07±0.10	0.01±0.01	0.30±0.29^*^
Cytoplasmic droplets (%)	0.02±0.02	0.15±0.11	0.02±0.01	3.04±2.18^*^
Clumped spermatozoa	0.25±0.31	0.96±0.77^*^	0.28±0.20	5.85±0.51^*^
Total abnormality	0.56±0.33	2.54±1.54^*^	0.65±0.27	14.83±2.57^*^

Results are expressed as Mean±SD. Means marked with * in the same row are significantly different from normal control for p<0.05, n=10. COLE: *Corchorus olitorius* leaf extract.

**Effects of COLE** **on reproductive hormones**

Serum concentration of FSH significantly increased in a dose-dependent manner following treatment with COLE in all test groups when compared with control (p<0.05). Luteinizing hormone concentration increased following treatment but declined with increasing dose of the extract. Estrogen concentration also increased steadily in the 250 and 500 mg/kg treatment groups before coming down drastically in the 1000 mg/kg treatment group. Testosterone concentrations in all test groups were significantly higher when compared with that of the control group (p<0.05). The values however declined with increasing dose of the extract (Table 5).

**Table 5:** Effects of COLE on reproductive hormones

**Parameters**	**Normal**	**COLE (250 mg/kg)**	**COLE (500 mg/kg)**	**COLE (1000 mg/kg)**
FSH (miu/ml)	7.16±0.24	7.70±0.98	9.16±1.18^*^	13.91±0.94^*^
LH (miu/ml)	4.18±0.24	45.71±5.50^*^	27.35±4.60^*^	12.16±1.60^*^
Estrogen E2 (ng/ml)	15.93±0.18	17.08±0.22^*^	20.46±0.46^*^	0.03±0.00^*^
Testosterone (ng/ml)	1.70±0.22	4.38±0.28^*^	4.26±0.12^*^	2.49±0.30^*^

Results are expressed as Mean±SD. Means marked with * in the same row are significantly different from normal control for p<0.05, n=10. COLE: *Corchorus olitorius* leaf extract.

**Effects of COLE on some reproductive organ histology**

**Effects on testicular histology**

**Figure 2:**
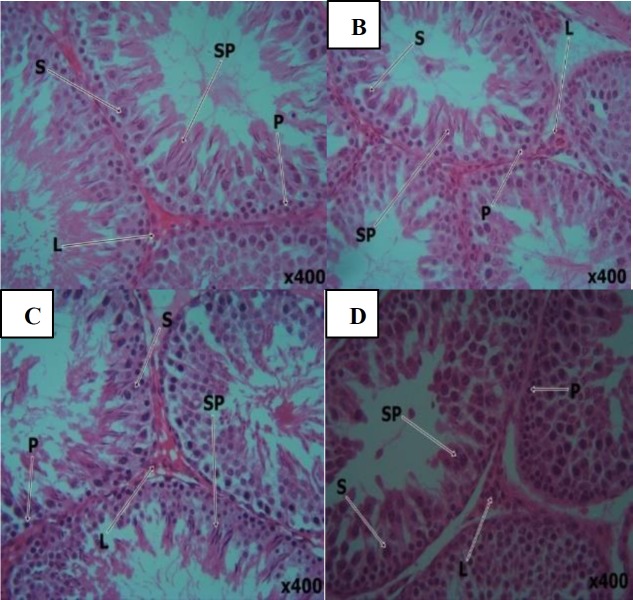
A) Control (H and E; X400). B) 250 mg/kg COLE treated (H and E; X400). C) 500 mg/kg COLE treated (H and E; X400). D) 1000 mg/kg COLE treated (H and E; X400)

Control photomicrograph of testes shows intact seminiferous tubules of uniform size with orderly germ cell maturation variable around the tubule, supported by the Sertoli cells. The mature spermatid density was variable in tubule and on average were 300 per tubule. The Leydig cells were also orderly differentiated. Testicular cross section of rats administered with 250 and 500 mg/kg of COLE, revealed no significant pathology but had average matured spermatid density of 200 and 280 per tubule respectively. Significant azoospermia was however observed in the testicles of rats administered with COLE 1000 mg/kg with an average matured spermatid density of about 30 per tubule (Figure 2A-D).

**Effect on** **histology of prostate**

**Figure 3:**
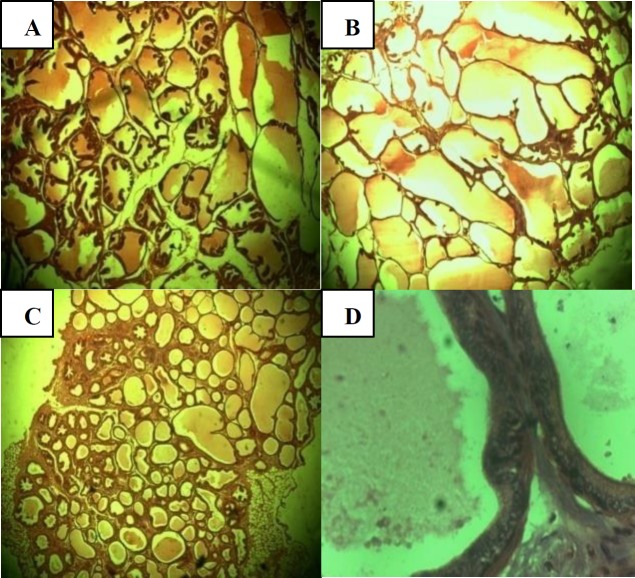
A) Control prostate, (H and E; X10)

The cross section of prostate histology in control showed active prostatic gland, containing corpora amylacea while in the 250 mg/kg treatment group, there was proliferation of glandular tissues which were tightly packed. In the 500 mg/kg treatment group, these proliferated glandular tissues were mostly seen to be composed of single layered epithelium which in the 1000 mg/kg treatment group was characterized by mitosis and prominent nucleoli (Figure 3A-D).

P=Spermatogonia, L=Interstitial cells (Leydig cells), S=Sertoli cell, and SP=Mature Spermatid.

B) 250 mg/kg COLE treated, (H and E; X40). C) 500 mg/kg COLE treated (H and E; X10). D) 1000 mg/kg COLE treated (H and E; X100).

S=Stroma (Connective tissue), G=Gland, CA=Corpora amylacea, SE=Squamous epithelium, and M=Mitosis.

## Discussion

The effects of *C. olitorious* leaves extract on sperm sample quality, hormone profile and testicular, epididymal and prostatic histology in male albino rats were investigated. The fall in body weight gains in test rats suggests that the extract may have interfered with the body’s fat depots, possibly via increased metabolism and decreased storage of fat and carbohydrate in the body. A combination of these processes has been implicated in the lowering of body weight gains (Ijioma et al., 2018). The extract in the gastrointestinal tract may have exerted inhibitory effects on the gastrointestinal smooth muscles leading to delayed transit of food substances and decreased appetite for additional food which ultimately may lower glucose available for body use and thereby affecting body weight due to compensation from alternative sources by gluconeogenesis. Although higher brain centers maintain body weight via energy homeostasis and modulation of appetite (Cummings et al., 2007), signals from the gastrointestinal tract (GIT) are key regulators of satiety and have implications for the control of body weights (Drucker, 2007). Inhibition of GIT motility suggests possible decline in the quantity of food consumed and may directly relate to the observed fall in body weight which corroborates with authors observation during the treatment period. Rats treated with COLE consumed lesser amounts of food when compared with food intake in the control group (Table 1). Most herbal medicines have excellent hypolipidemic actions without side effects (Vijayakumar et al, 2018). We had shown in our previous communication that COLE exhibited significant hypolipidaemic activity following trials in Wistar rats (Orieke et al., 2018), and may have been responsible for the observed decline in body weight gains in the treated rats. Plant extracts with hypolipidemic effects are known to increase breakdown of lipids accumulated in adipose tissue leading to fall in body weight gain (Omodamiro et al., 2016). This effect of the extract appears to agree with a local claim on its use by pregnant women in Nigeria, to control fetal weight and enhance easy delivery process (Personal Communication). This however requires further evaluations.

The presentation of organ weight data relative to the animal’s body weight may help to remove bias due to different body weights (Ijioma et al., 2018). Indeed organ weight is one of the major indicators of the effect of a test substance because significant differences in organ weights among test and control animals may occur in the absence of observable morphological changes (Ramakrishna et al., 2015). The fall in testicular weight in rats treated with the highest dose of COLE may be attributed to the fall in their testosterone levels. Testosterone plays role in the enhancement of testicular growth, hence, severe declines in its concentration may cause testicular atrophy (Ogwo et al., 2016). 

Sperm sample color did not significantly differ among test groups and control, suggesting that treatment with the extract did not alter sperm sample color which expectedly should be creamy for better reproductive performance. Sperm sample pH is an important parameter in the evaluation of sperm sample quality and is a well-known determinant of the survival rate of sperm cells. Acidic media, whether in the female vagina or sperm sample itself, are known to increase sperm mortality, and inhibit sperm performance and conception and are among the causes of infertility (Zhoun et al., 2015). Alkaline medium is therefore the most ideal environment for sperm performance since it neutralizes the acidity of the female vagina and presents an environment which promotes optimum sperm motility and enhances the processes leading to fertilization of ovum (Guyton and Hall, 2006). Therefore, while the alkaline semen environment observed in the lowest dose treatment group suggests that the dose favors reproduction in rats, the low pH observed in the rats treated with 1000 mg/kg may correlate with inhibitory effect on both sperm survival and reproduction. It is also important to note that in this treatment group, individual sperm motility, progressiveness and viability all were lower than that of the control group, further confirming that administration of high dose *C. olitorius* extract may negatively affect reproductive performance in males. Recent studies and reports have linked progressive motile movement of sperms with high fertility index (Love, 2011). Sperm motility was reported to be enhanced by the fructose content of sperm samples and at the highest dose used, the extract may have lowered sperm sample’s fructose content leading to fall in motility and viability of the sperm cells. These findings appear to agree with data reported by Oyedeji et al. (2013), that the consumption of high amounts of *C. olitorius* extract produced negative effects on the reproductive systems of male rats. The work of Oyedeji et al. (2013) was however limited to analysis of few sperm sample parameters, prostrate and testicular histology of a single dose level treatment; therefore, it did not adequately represent actual reproductive functions of the test rats. The fall observed in sperm sample concentration following treatment of the male rats with the extract, particularly at 1000 mg/kg, further corroborates with earlier assertions that the extract may have affected the male reproductive system negatively. Testicular weights in this treatment group diminished greatly and may have accounted for the observed fall in sperm sample concentration. In the testes, sperm cells are formed in the seminiferous tubules and atrophy in this vital testicular chamber, decreased spermatids, diminished fertility (Cheah and Yang, 2011), troubled spermatogenesis and decreased reproductive performance (Ahmed-Farid et al, 2017). This may also account for the observed increase in the percentage of abnormal sperm cells formed in this treatment group. Atrophy of seminiferous tubule may have reduced the quantity of raw materials needed for sperm formation in addition to limited space which eventually affects the number of sperms formed and increased the chances of head, mid piece and tail abnormalities in the produced sperm cells. Increases in sperm abnormalities generally can impair motility (tail abnormality), energy supply (mid piece abnormality) and ability to achieve fertilization (head abnormality). The relationship between sperm abnormalities and infertility is well established (Guyton and Hall, 2006). The mechanism through which administration of high-dose *C. olitorious* leaves extract causes increases in the number of abnormal sperm cells, requires further evaluation.

The increase in follicle stimulating hormone (FSH) concentration following treatment with the extract suggests that the extract may have improved reproductive function in the treated male rats. In males, FSH regulates sexual development, growth, pubertal maturation and reproductive qualities by enhancing the induction and maintenance of normal sperm production (Vasudevan et al, 2011; Simoni et al., 1999). However, excessive increase in FSH concentration as observed in the 1000 mg/kg treated rats may negatively affect reproduction due to its effect on spermatogenesis. It was established that the most common endocrine abnormality associated with male infertility or sub-fertility is elevated FSH concentration, which generally indicates the impairment of spermatogenesis and primary testicular defect (Hideyuki et al, 2012).

The concentrations of testosterone and luteinizing hormones in the lower doses treated rats increased but declined in the high-dose group, suggesting that administration of high-dose extract may be deleterious to the male reproductive system. Luteinizing hormone promotes male reproductive functions by stimulating testosterone production from Leydig cells in the testis (Sembuligam and Prema, 2016; Payne and O’shaughnessy, 1996) and may be the reason for the increase in testosterone concentration in the low and moderate dose treated groups. As a primary sex hormone, testosterone promotes the development of the reproductive organs and secondary sexual characteristics in males (Sembuligam and Prema, 2016). The observed decline in testosterone concentration following high-dose treatment may have resulted from either the direct effect of the extract on the Leydig cells or indirectly as a consequence of decreased luteinizing hormone level in the group. Decline in testosterone concentration is reportedly a major sign of infertility (Okereke and Onuoha, 2015). The fall in estrogen concentration in the rats treated with 1000 mg/kg body weight of the extract may be due to the observed atrophy of the testes of the rats which may have caused severe reduction in the amount of adipose tissues in the testicles of the treated rats. Estrogen in males is secreted by the adrenal glands and testes and increases with increasing quantity of body fat. This may explain why overweight men are more commonly affected by high estrogen levels (Cabler et al., 2010). The increase in estrogen concentrations in the groups treated with lower doses also suggests that the extract in males may have anti-fertility effects. High estrogen concentrations in males have greatly been associated with infertility due to its sperm count lowering effect (Cabler et al., 2010)

Histological sections of the testes of rats treated with the extract showed no significant pathology at lower dose but with fall in sperm density when compared with control and testicular atrophy in the group administered with COLE 1000 mg/kg. The reduction in sperm load and testicular atrophy following treatment with COLE 1000 mg/kg may be due to the observed decline in the concentration of testosterone following treatment. Findings from this work appear to agree with those reported by Oyedeji et al. (2013) indicating that treatment of male rats with aqueous extract of *C. olitorius* leaves extract decreased testosterone levels, sperm motility, sperm count, and sperm viability but increased the percentage of abnormal sperm cells (with distortion of testicular structure) relative to the control rats. These researchers may have administered high doses of the extract to the experimental rats. 

If the results obtained in this work carried out in rats, can be extrapolated to men, we conclude that the consumption of high amounts of *C. olitorius* may inhibit reproductive functions in males due to decreased semen quality, increased sperm abnormalities and estrogen levels, atrophy of testicles and negative changes affecting both the histological architecture and structural organization of the testes in the treated animals.
